# Translation-Independent Roles of RNA Secondary Structures within the Replication Protein Coding Region of Turnip Crinkle Virus

**DOI:** 10.3390/v12030350

**Published:** 2020-03-22

**Authors:** Rong Sun, Shaoyan Zhang, Limin Zheng, Feng Qu

**Affiliations:** Department of Plant Pathology, The Ohio State University, Wooster, OH 44691, USA; sun.1207@buckeyemail.osu.edu (R.S.); zhang.5635@buckeyemail.osu.edu (S.Z.); zheng.1811@osu.edu (L.Z.)

**Keywords:** plant virus, positive-sense RNA virus, RNA secondary structure, translational read-through, replication

## Abstract

RNA secondary structures play diverse roles in positive-sense (+) RNA virus infections, but those located with the replication protein coding sequence can be difficult to investigate. Structures that regulate the translation of replication proteins pose particular challenges, as their potential involvement in post-translational steps cannot be easily discerned independent of their roles in regulating translation. In the current study, we attempted to overcome these difficulties by providing viral replication proteins in *trans*. Specifically, we modified the plant-infecting turnip crinkle virus (TCV) into variants that are unable to translate one (p88) or both (p28 and p88) replication proteins, and complemented their replication with the corresponding replication protein(s) produced from separate, non-replicating constructs. This approach permitted us to re-examine the p28/p88 coding region for potential RNA elements needed for TCV replication. We found that, while more than a third of the p88 coding sequence could be deleted without substantially affecting viral RNA levels, two relatively small regions, known as RSE and IRE, were essential for robust accumulation of TCV genomic RNA, but not subgenomic RNAs. In particular, the RSE element, found previously to be required for regulating the translational read-through of p28 stop codon to produce p88, contained sub-elements needed for efficient replication of the TCV genome. Application of this new approach in other viruses could reveal novel RNA secondary structures vital for viral multiplication.

## 1. Introduction

Viruses with single-stranded (ss) positive-sense (+) RNA genomes harbor various intra-genome RNA secondary structures and sequence motifs that play critical *cis*-acting roles in their infection cycles [1]. Among the best-known virus-encoded RNA secondary structures are the internal ribosomal entry site (IRES) elements found in many viruses that enable efficient translation of viral proteins by guiding ribosomes directly to the start codon [2]. Different RNA sequence motifs or structures within the same or different viral genomic RNA (gRNA) are also known to engage in long-distance interactions in order to enhance the translation of viral genes, or to facilitate the synthesis of viral subgenomic RNAs (sgRNAs) [1,3,4]. Additionally, many internally encoded stem–loop structures have been shown to exert diverse functions, including serving as the binding sites for viral RNA-dependent RNA polymerase (RdRP), as well as the initiation site of genome encapsidation [3,5].

Despite their well-recognized roles in viral multiplication cycles, some of the RNA secondary structures are difficult to study because they often reside in coding sequences for important viral proteins, including RdRPs and auxiliary replication proteins (ARPs). For instance, many a (+) RNA virus encode RdRP by extending ARP at the C-terminus by avoiding the stop codon of the latter through either translational read-through or frame-shifting. Such read-through and frame-shifting events are tightly regulated through highly conserved RNA structures that simultaneously encode part of the RdRP [6,7]. For RNA structures with roles in an early step of viral replication cycle, e.g., translation of ARP or RdRP, it is difficult to discern whether they also participate in later steps, like genome replication or assembly, because perturbing the early steps would in turn interfere with later steps.

**Figure 1:**
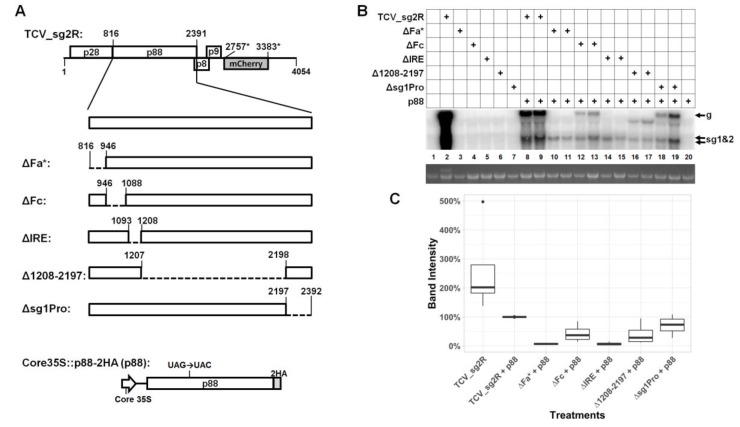
Deletions within the p88 coding sequence differentially affect the abundance of TCV gRNA in *N. benthamiana* cells. (**A**) Schematic representation of five in-frame deletions within the p88 coding sequence. The top diagram depicts the genome of TCV_sg2R, a derivative of TCV encoding mCherry in place of TCV CP. It is the backbone for all deletion and point mutants used in this study. The numbers are nucleotide positions in TCV gRNA. Dashed lines represent the regions deleted. (**B**) Results of a representative Northern blotting, showing the accumulation levels of different deletion mutants in the presence of trans-supplied p88. None of the mutants could replicate on their own. (**C**) Quantification of the gRNA accumulation levels of the deletion mutants. The box plots were based on relative intensity of the gRNA bands, derived from four repeats. The band intensity of (TCV_sg2R + p88) was set as the reference for comparison.

Here we report an approach to uncouple the protein-coding capacities of viral gRNA from the RNA secondary structures it folds into, using turnip crinkle virus (TCV) as our model. TCV is a small (+) RNA plant virus that counts model plants Arabidopsis and *Nicotiana benthamiana* as hosts [7,8,9,10,11]. Its genome of 4054 nucleotides (nt) encodes five proteins (Figure 1A). The 5′ proximal p28 and its read-through product (p88) are TCV-encoded ARP and RdRP, respectively, both needed for genome replication. The p8 and p9 movement proteins (MPs), and p38 capsid protein (CP), are translated from two sgRNAs produced during viral replication (Figure 1A). CP is also the suppressor of RNA silencing-mediated host defense. However, it is not required for genome replication as long as the activity of RNA silencing is kept in check with a heterologous suppressor, such as p19 of tomato bushy stunt virus (TBSV) [8,9,12].

Both p28 and p88 are translated directly from TCV gRNA, with the latter resulting from programmed translational read-through of the p28 stop codon. This translational read-through event is facilitated by a well-characterized, highly conserved RNA secondary structure known as a recoding stimulatory element (RSE), in coordination with another 3′ terminal structure through a long-distance kissing loop interaction [6,7]. RSE also contains two internal stretches of sequences that interact with each other to form a pseudoknot structure necessary for its role in stimulating translational read-through [7]. Earlier studies suggested that some of the sequences within RSE might also be important for TCV genome replication [13]. However, this could not be easily examined because lower genome replication could also be caused by a lack of p88 resulting from RSE mutations that diminish the read-through translation.

In the current study, we assessed the translation-independent role of several previously identified structure/sequence elements within p28/p88 coding sequence by providing the p28/p88 proteins in *trans*, thus obviating the need to maintain the protein-coding capacity and the structures that regulate protein translation within the p28/p88 coding region of TCV genome. This alternative approach allowed us to confirm a replicational role of a previously characterized structure, known as internal replication element (IRE) [5]. More importantly, it permitted us to uncover a novel role of several RSE-resident structural elements in TCV gRNA accumulation. This novel approach should be easily adaptable to other viral RNAs, leading to further assessment of many known RNA structures and the identification of new structures.

## 2. Materials and Methods

### 2.1. Constructs

The constructs TCV_sg2R, Core35S::p88-2HA, and 2 × 35S::p28 (tag-free) were described in previous studies [10,12,14]. All of the new mutant replicons, including 813UAA, p28TS, and the deletion mutants, as well as other mutants with point mutations, were made on the TCV_sg2R backbone. The mutations were generated with either overlapping PCR with appropriate primers or with mutation-containing gBlock fragments synthesized by Integrated DNA Technologies. The identity of all new constructs was verified with Sanger sequencing.

### 2.2. Agro-Infiltration

Upon verification, the constructs were introduced into *Agrobacterium tumefaciens* strain C58C1 with electroporation [9]. In most experiments, various combinations of *Agrobacterium* suspensions were mixed and delivered into *N. benthamiana* leaves, as described in [9,12,14]. A p19-expressing *Agrobacterium* strain was included in all combinations to alleviate RNA silencing-mediated mRNA degradation.

### 2.3. RNA Extraction and Northern Blotting

Total RNA was extracted from agro-infiltrated *N. benthamiana* leaves, using the Direct-zol RNA Miniprep kit (Zymo Research, Irvine, CA, USA). To ensure consistency, four equivalent leaf sections derived from infiltrated leaves of four different plants were pooled before RNA extraction. The RNA was then quantified with NanoDrop and subjected to Northern blotting, as described in [12,14]. Quantification of Northern blotting results was carried out with ANOVA.

## 3. Results

### 3.1. Much of the p88 Coding Sequence is Dispensable for TCV gRNA Accumulation in Infected Cells

To assess the role of RNA secondary structures or conserved sequence motifs within the p88 coding region of TCV independent of the RdRP function of the p88 protein, we supplied p88 in *trans*, using a transient expression construct, Core35S::p88-2HA (Figure 1A, bottom). The low-level p88 production from this construct, driven by the Core35S promoter (the last 99 nucleotides (nt) of the cauliflower mosaic virus 35S promoter), was shown previously to complement the replication of a p88-defective mutant TCV replicon [10]. This construct was delivered into the cells of *Nicotiana benthamiana* leaves, along with a series of mutant TCV replicons containing deletions within the C-terminal 2/3 of p88 open reading frame (ORF), leaving the coding sequence of p28 intact (Figure 1A). The deletions were based on TCV_sg2R, a TCV replicon encoding the mCherry red fluorescent protein in place of p38 [8,9]. Accordingly, TBSV p19 was included in all treatments, to counteract RNA silencing. Altogether, five in-frame deletions were generated, encompassing Fa, Fc, and IRE, three RNA elements reported in various previous studies [5,13,15], a 990-nt (positions 1208 to 2197) region with no known structural features, and the promoter of sgRNA1 (sg1Pro) [16]. The Fa element is also part of the well-characterized RSE structure, controlling translational read-through of the p28 ORF to produce p88 [1,7] (also see later).

An additional control included in nearly all experiments was the mix of trans-supplied p88 and the TCV_sg2R replicon encoding its own p88 (Figure 1B, lanes 8 and 9). This is important because p88, even at the low levels permitted by the Core35S promoter, exerted both repressive and complementing roles on the co-delivered TCV replicons, making TCV_sg2R without p88 in *trans* an inadequate positive control [10] (also see Figure 1B; compare lane 2 with lanes 8 and 9). As expected, without p88 provided in *trans*, none of the five mutant replicons produced viral gRNA to levels detectable by Northern blotting (Figure 1B, lanes 3–7). In contrast, the presence of p88 enabled three mutants, ΔFc (lanes 12 and 13), Δ1208–2197 (lanes 16–17), and Δsg1Pro (lanes 18–19) to accumulate their corresponding gRNAs to easily detectable levels (approximately 50%–80% of TCV_sg2R plus p88; Figure 1C). Therefore, the regions deleted in these mutants, 141, 990, and 195 nt in respective lengths, were unlikely to contain *cis*-acting elements indispensable for TCV gRNA replication.

### 3.2. Two Short Sections of the p88 Coding Sequence Are Essential for Robust Accumulation of TCV gRNA but not sgRNA

By contrast, the ΔFa* and ΔIRE deletions, 129 and 114 nt respectively, diminished the viral gRNA levels to below the detection limit of Northern blotting (Figure 1B, lanes 10 and 11; 14 and 15). The requirement of IRE for TCV replication was previously investigated by others [5]. Nevertheless, we were surprised to find that the ΔIRE mutant still produced sgRNAs to levels comparable to the ΔFc, Δ1208–2197, and Δsg1Pro mutants (Figure 1B). Therefore, in the presence of trans-supplied p88, the IRE appeared to be needed only for the accumulation of TCV gRNA. Similarly, the Fa element (nt 816–847, the first 32 nt of the ΔFa* deletion), as part of RSE, was previously found to be essential for the read-through translation of p88 [7]. However, the read-through requirement of Fa would have been released by the trans-supplied p88 in our system. Thus, the fact that the ΔFa* mutant also abolished gRNA accumulation suggested the existence of a read-through-independent element within the deleted region that is required for TCV gRNA accumulation. Similar to the IRE, this novel function of Fa appeared to have minimal effects on sgRNA accumulation. Hence, the activities of IRE and Fa both appear to be gRNA-specific.

### 3.3. An Eight Base-Pair (bp) Stem within the Lower Half of RSE Modestly Contributes to TCV gRNA Accumulation

**Figure 2:**
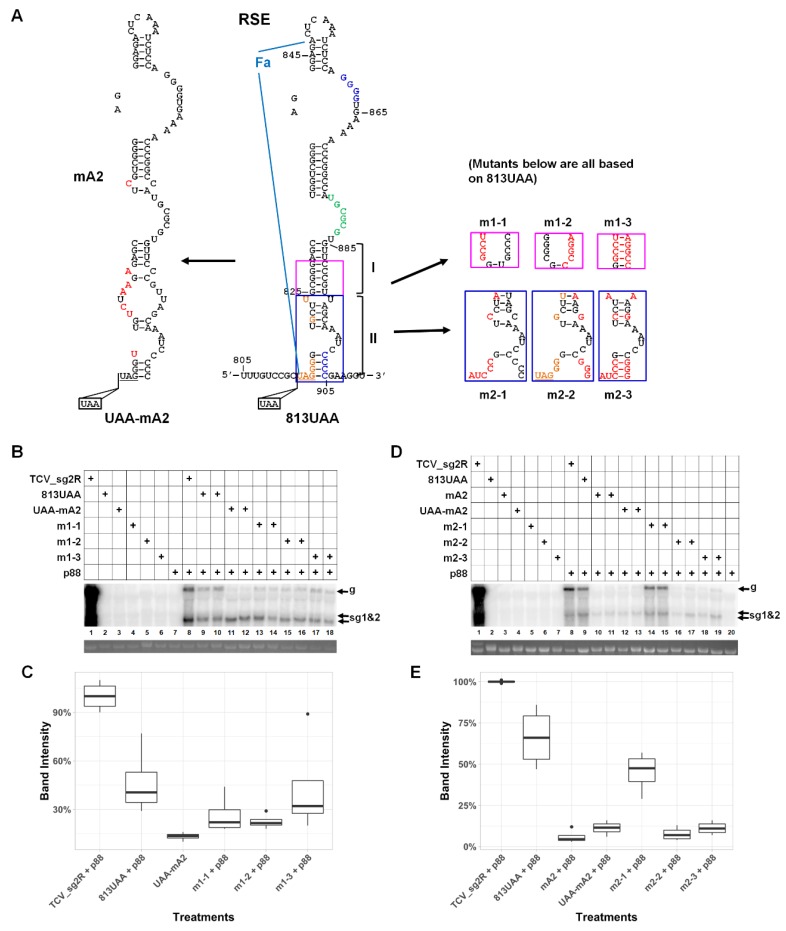
Role of the Fa and RSE in TCV gRNA accumulation. (**A**) Diagrams of all mutants tested in this set of experiments. The RSE secondary structure is shown in the middle. The boundaries of the Fa element are highlighted to the left with two blue lines. The seven highly conserved nts at the beginning of the element are in orange letters. The CCCC and GGGG stretches implicated in an intra-RSE pseudoknot are in bright blue letters. The six nts implicated in a long-distance interaction, UGCGCG, are in green letters. The lower half of the RSE, which was the main focus of the current study, is further divided in stems I and II, to simplify the narration. The pink and bright blue boxes highlight the sections being investigated with mutagenesis. Most of the mutants also contained the UAA insertion shown at the bottom. The left-side diagram shows all the nts mutated in the mA2 mutant. (**B**) Northern blotting revealed the impact of 813UAA, mA2, m1-1, m1-2, and m1-3 mutants on TCV gRNA levels. (**C**) Box plot quantification of Northern blotting results in (**B**), with multiple repeats. (**D**) Northern blotting revealed the impact of m2-1, m2-2, and m2-3 mutants on TCV gRNA levels. Note that the m2-1 mutant mutated all of the seven conserved nts and was expected to extensively perturb stem II. (**E**) Box plot quantification of Northern blotting results in (**D**), with multiple repeats.

We next set out to identify potential RNA motifs and/or structures responsible for the diminished gRNA accumulation in the ΔFa* mutant. Importantly, the ΔFa* deletion (nt positions 817–946) encompassed two previously reported structures—Fa (nt 816–847) and RSE (nt 816–905), with the former being the front half of the latter [7,13,15] (Figure 2A, middle drawing; the boundaries of Fa are highlighted with light blue lines). Therefore, we first interrogated RSE by reexamining the previously reported mA2 mutant [13]. As shown in Figure 2A (left diagram, red letters denote mutated nts), the mA2 mutant contained seven point mutations causing extensive disruption of the lower half of the RSE structure. To further eliminate the impact of translational read-through, we created a new 813UAA mutant by inserting a second stop codon (UAA) in front of the original UAG stop codon of p28 (Figure 2A). This 813UAA construct served as the backbone for all site-specific mutants described hereafter. A UAA-mA2 mutant was also created by combining the mA2 mutations with the 813UAA insertion.

In the presence of p88 in *trans*, the gRNA levels of the 813UAA mutant were 40–70% of TCV_sg2R in different experiments (Figure 2B, lanes 8–10; Figure 2D, lanes 8 and 9; and Figure 2C,E for quantifications), which probably reflected a small advantage afforded by the *cis*-translated p88 by TCV_sg2R. Nevertheless, the mA2 mutations, with or without the extra UAA mutation, caused the corresponding gRNAs to decrease to approximately 10% of TCV_sg2R levels, or close to 20% of the 813UAA levels (Figure 2B, lanes 11 and 12; Figure 2D, lanes 10–13). Therefore, the mA2 mutations, by disrupting the lower stem of RSE, caused a five-fold loss of TCV gRNA levels independent of translational read-through. These results suggested that the Fa region of the RSE element also played important roles in TCV genome accumulation.

Six of the seven mutations in the mA2 mutant are within the lower half of RSE, underneath the 7-nt asymmetric loop in green (Figure 2A). To further delineate the area needed for TCV gRNA accumulation, we next introduced mutations within stem I, the 8-bp G/C-rich stem immediately below the asymmetric loop (Figure 2A; only the base pairs in the pink box were mutated). As shown in Figure 2B, disrupting stem I with either m1-1 or m1-2 (Figure 2A, the pink boxes) caused a modest decrease of viral gRNA levels (approximately 50% of 813UAA) that was less severe than mA2 (20%). Importantly, this decrease was notably mitigated when the base-pairing was restored with m1-3, which combined the mutations in m1-1 and m1-2 (Figure 2B, lanes 13–18). These results indicated that maintenance of the stem I base pairs, but not the nt identities, plays a moderate role in TCV gRNA accumulation. Again, the sgRNA levels were conspicuously unaffected.

### 3.4. A Highly Conserved Sequence Motif in the Vicinity of p28 Stop Codon Contributes Little to TCV gRNA Abundance

We then interrogated stem II, located at the bottom of RSE, to assess its potential impact on TCV gRNA accumulation. Earlier studies identified two key features within this portion of RSE: (i) The first 11 positions of the RSE contain seven invariable nts highly conserved among more than 35 viruses of *Tombusviridae* (UAGGGGUGCUU, the underlined nts are invariable; also see Figure 2A, bottom-left portion of RSE, for nts in orange) [7]; (ii) the four Cs on the right side of stem II (Figure 2A, blue letters), which pair with four Gs 39-nt upstream (also in blue letters in the RSE diagram) to form a pseudoknot, are crucial for efficient translational read-through [7]. However, the potential roles of these two features in TCV gRNA accumulation were not investigated.

To determine whether the seven conserved nts contribute to TCV gRNA levels in infected cells, we created m2-1, in which all of the conserved nts were mutated into their complementary residues (Figure 2A, lower right, first blue box; red letters denote the mutated nts). Surprisingly, the gRNA of this mutant accumulated to approximately 70% of the 813UAA levels (Figure 2D,E). Therefore, mutating all of the seven conserved nts caused only a minimal loss of TCV gRNA accumulation; hence, their conservation appears to be only crucial for translational read-through. Importantly, the m2-1 mutant also caused extensive disruption of stem II, especially the four base pairs at the bottom of RSE. Therefore, the integrity of stem II likely has negligible contribution to TCV gRNA accumulation as well.

### 3.5. The Lower-Right Side of Stem II Plays a Dominant Role in TCV gRNA Accumulation

Mindful of the previous study showing that the CCCC motif at the bottom-right of RSE engaged in a stable pseudoknot with GGGG near the top of RSE (nt 860–863)—and this pseudoknot was needed for efficient translational read-through [7]—we next created mutant m2-2 by mutating three of these four Cs, plus two additional mutations slightly above. These mutations, if combined with the m2-1 mutations, were expected to restore the RSE secondary structure (Figure 2A, second and third blue boxes). Surprisingly, m2-2 gRNA accumulated to just 20% of 813UAA, a level similar to mA2 (Figure 2C, lanes 16 and 17). Furthermore, restoring the base-paired state for this portion of RSE (mutant m2-3) failed to mitigate the defect caused by m2-2 mutations (Figure 2C, lanes 19 and 20). These results hinted that the previously identified pseudoknot might also be needed for efficient TCV gRNA accumulation.

### 3.6. Robust Accumulation of TCV gRNA Depends on the Integrity of a Previously Identified Pseudoknot within RSE

**Figure 3:**
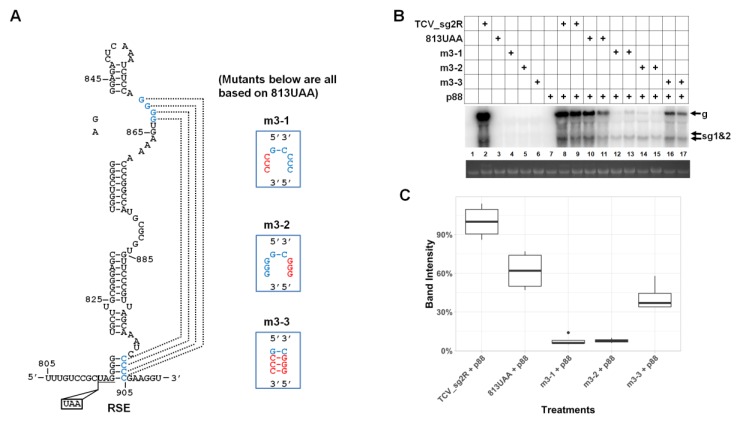
The intra-RSE pseudoknot has a read-through-independent role in TCV gRNA accumulation. (**A**) The intra-RSE pseudoknot. The nts implicated in the pseudoknot are highlighted in blue letters, with the anticipated base-pairing relationship denoted with dotted lines. Three mutants tested, with m3-1 and m3-2 disrupting the pseudoknot, and m3-3 restoring the pseudoknot by combining the mutations in m3-1 and m3-2, are also depicted. (**B**) A representative Northern blotting showing accumulation levels of the pseudoknot mutants. (**C**) Box plot quantification of Northern blotting results from multiple repeats.

The results with mutants m2-2 and m2-3 suggested that the specific nt sequence altered in m2-2 (Figure 2A) was crucial for robust TCV gRNA accumulation. This result is reminiscent of the finding by Kuhlmann and colleagues [7], because among the nts altered in m2-2 (and m2-3) were three of the four Cs implicated in the pseudoknot they discovered. We hence tested whether this pseudoknot was indeed also important for TCV gRNA accumulation, when the need for translational read-through was obviated with trans-supplied p88. As shown in Figure 3, mutating three of the four Cs alone was sufficient to cause a reduction of TCV gRNA level to less than 20% of the 813UAA control (Figure 3B, mutant m3-2, lanes 14 and 15). Strikingly, mutating three of the four Gs that formed the other strand of the pseudoknot caused a TCV gRNA reduction to the similar extent (lanes 12 and 13). By contrast, restoring the base-pairing to the pseudoknot was enough to recover the gRNA level to approximately 70% of the 813UAA control (Figure 3B, lanes 16 and 17; Figure 3C). In conclusion, the GGGG/CCCC pseudoknot is required for both translational read-through and TCV gRNA accumulation.

### 3.7. The Long-Distance Kissing Loop Interaction between RSE and the 3′ Terminal Stem–Loop does not Contribute to TCV gRNA Abundance

**Figure 4:**
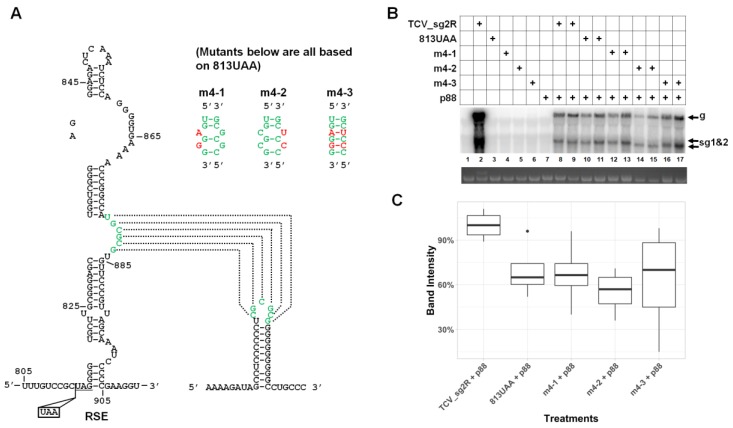
The long-distance kissing-loop interaction does not have a read-through-independent role in TCV gRNA accumulation. (**A**) Diagrams depicting the kissing-loop interaction between the asymmetric loop in RSE and the end loop of the 3′ terminal stem–loop of TCV gRNA, and the mutants tested in this set of experiments. The specific nt changes in these mutants are identical to those in a previous study (see text for details). (**B**) A representative Northern blotting showing accumulation levels of the mutants. (**C**) Box plot quantification of Northern blotting results.

The reductions in TCV gRNA levels by the mA2 mutations, as well as the pseudoknot mutations, while substantial, were short of the near complete loss of the ΔFa* mutant. We hence moved to test another known sequence motif inside RSE, namely the asymmetric loop roughly in the middle of RSE (Figure 4A, green letters) and the long-distance kissing loop interaction it partakes, for their role in TCV gRNA accumulation. For this, we introduced the same mutations reported by Cimino and colleagues [6] into our 813UAA backbone (Figure 4A, m4-1 to m4-3). As shown in Figure 4B, the gRNAs of all three mutants accumulated to levels similar to the 813UAA parent. Therefore, unlike the pseudoknot examined earlier, this long-distance interaction appears to be needed solely for translational read-through.

### 3.8. A Sequence Element within p28 Coding Sequence has a Modest Role in TCV gRNA Accumulation

**Figure 5:**
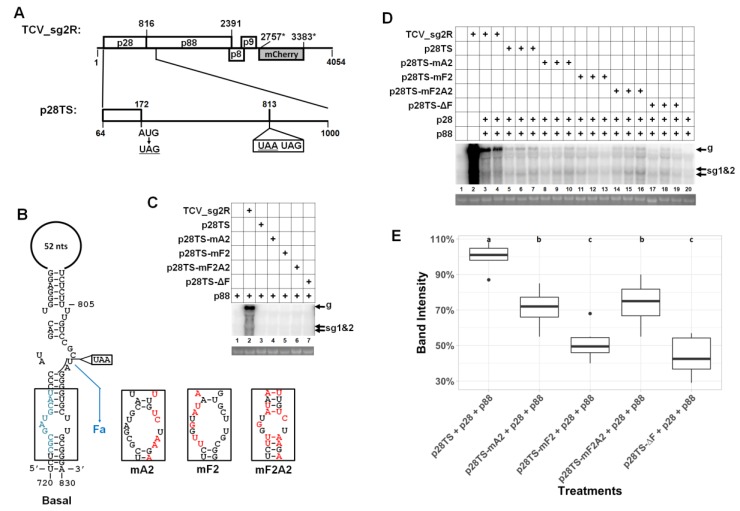
Interrogation of an RNA element within the p28 coding sequence by providing both p28 and p88 in *trans*. (**A**) Schematic depiction of the p28TS mutant replicon encoding neither p28 nor p88, with the positions of three stop codons shown. (**B**) The Basal RNA structure involving mostly p28 coding sequence. Note that this structure is mutually exclusive with RSE because, in this structure, the Fa element pairs with the Ff element (in blue letters) within the p28 coding sequence. Accordingly, the mA2 mutations shown in Figure 2 now disrupt the FfFa stem structure. Two additional mutants, mF2 and mF2A2, were also tested, along with a Ff deletion mutant (ΔF). All mutants were based on p28TS. (**C**) Northern blotting showed that, unlike earlier mutants, these mutants did not replicate in the presence of trans p88 alone. (**D**) Northern blotting revealed a modest role of Ff in TCV gRNA accumulation. (**E**) Quantification of Northern botting results of multiple repeat experiments.

Aside from the Fa, RSE, Fc, IRE, and sg1Pro elements described earlier, a previous study also reported another element, known as Ff, located within the coding sequence of p28 [15]. The Ff element was predicted to interact with Fa to form another stem–loop structure designated as “Basal” (Figure 5B), and the Basal structure is mutually exclusive with RSE [7,13]. We hence attempted to examine the potential role of Ff in TCV gRNA accumulation, by providing both p28 and p88 in *trans*. For this purpose, we first created another control replicon, designated p28TS (TS = triple stops), by changing the codon position #37 of p28 from AUG to UAG, in the 813UAA background (Figure 5A). This p28TS replicon was then used as the backbone for mA2 to create p28TS-mA2, as well as three new mutants: p28TS-mF2, -mF2A2, and -ΔF. The last mutant had the entire Ff element deleted (Figure 5B, lower left, grayish-blue letters).

None of the new mutants could accumulate detectable levels of gRNA in the presence of p88 alone (Figure 5C), illustrating that, unlike TMV [17], the TCV-encoded p28 ARP was absolutely needed for genome replication. The p28TS replicon did accumulate gRNA to detectable levels in the presence of both p28 and p88 (Figure 5D, lanes 5–7). However, this accumulation was 50% lower than the 813UAA/p88 combination. Interestingly, introducing the mA2 mutations into p28TS caused a very modest further reduction of viral gRNA levels (70%; Figure 5D, lanes 8–10; Figure 5E), suggesting that the role of Fa in replication might be coupled to *cis*-produced p28. Nevertheless, the mutations in mF2, which were predicted to disrupt the lower stem of the Basal structure depicted in Figure 5B, did cause a 50% reduction in gRNA levels relative to p28TS (Figure 5D, lanes 11–13; Figure 5E). Furthermore, this reduction appeared to be alleviated by restoring the disrupted base-pairing through the combination of mA2 and mF2 mutations (Figure 5D, lanes 14–16). Finally, deleting the Ff element caused a reduction in viral gRNA levels comparable to mF2. Collectively, these results suggest that both p28 and p88 could be provided in *trans*, to facilitate the replication of TCV gRNA, but the complemented replication was less robust when compared to replicons that encode both proteins in *cis*. Additionally, the previously identified Ff element appeared to modestly stimulate TCV gRNA accumulation, probably through the “Basal” stem–loop structure stabilized by the Ff/Fa interaction.

## 4. Discussion

We report here further examination of RNA secondary structures embedded in the coding sequence of two TCV replication proteins, p28 and p88. Some of these structures were previously shown to be essential for the translational read-through of the p28 stop codon in order to synthesize the p88 RdRP [6,7]. However, it was unclear whether they had additional roles in TCV multiplication independent of translational read-through. Such roles were not easy to discern because mutations that disrupt the RNA structures needed for translational read-through (e.g., RSE) would block the translation of p88, hence abolishing replication, even if all RNA structures needed for replication remained undisturbed. Our alternative approach sought to provide p88, and later on, p28 as well, from separate replication-independent sources, in an attempt to bypass the need for translational read-through. This approach allowed us to make several important observations.

First, more than 1/3 of the 2328-nt p88-coding sequence can be removed without substantially compromising the replicability of the TCV genome relative to appropriate controls. This is evident from the notable replication levels of three deletion mutants: ΔFc (deleting 141 nt), Δ1208–2197 (deleting 990 nt), and Δsg1Pro (deleting 195 nt). Although the gRNA accumulation levels of these mutants were modest in comparison to the TCV_sg2R + p88 control, they were similar to another control replicon (813UAA + p88) incapable of producing any p88 by itself, suggesting a slight advantage of *cis*-produced p88. Second, production of TCV sgRNA does not always depend on the synthesis of gRNA. In the presence of p88 in *trans*, two deletion mutants, ΔFa* and ΔIRE, as well as several other mutants, failed to produce detectable levels of gRNA but exhibited normal levels of sgRNA. This observation indicates that minus-strand RNA synthesis is still active under these conditions, as sgRNA-sized minus-strands are required as templates for sgRNA transcription [18]. Thus, the deleted RNA elements could be required for (i) completion of full-length minus-strand synthesis and/or (ii) efficient initiation and synthesis of the full-length plus-strands from a full-length minus-strand template. The uncoupling of sgRNA transcription from genome replication based on modifications to the RdRP has been reported for tombusviruses [19]. Additionally, the synthesis of gRNA and sgRNA may occur in different microenvironments. This is consistent with recent observations of different sized replication organelles in cells replicating flock house virus that correspond to the different sized genomic and subgenomic RNAs [20]. Conversely, additional *cis*-acting elements in gRNA could allow replication proteins to discriminate against faulty templates.

Third, structures implicated in translational read-through can have additional roles in genome replication. This is best illustrated by the pseudoknot structure residing in the RSE of TCV, which was found earlier to be needed for efficient translational read-through. We found that it was also needed for efficient accumulation of TCV gRNA, probably through its participation in genome replication. It is possible that this dual function could be related to coordinating translational read-through with genomic minus-strand synthesis, which are directionally opposing processes on the genomic RNA. In this scheme, read-through would unfold the pseudoknot needed for full-length genome minus-strand synthesis, thereby inhibiting this competing process. The maintenance of sgRNA accumulation when the pseudoknot is disrupted suggests that the inhibition may be specific for synthesis of full-length genomic minus-strands. RNA-mediated coordination of these two processes occurs in tombusviruses, where formation of the long-range interaction between the RSE and 3′ UTR required for RdRP read-through concomitantly prevents formation of an alternative RNA structure in the 3′UTR needed for minus-strand RNA synthesis [6]. More detailed studies are required to address possible regulatory effects of the RSE in TCV.

The integrity of the middle portion of the RSE (stem I) may also serve as an additional sentinel for quality control of TCV genome. It is possible that involvement of stem I in TCV replication could entail collaboration with p28 translated from the same RNA, because the reduction in gRNA levels caused by mA2 mutations was much less pronounced in the p28TS replicon backbone (in need of both p28 and p88 in *trans*) than in the 813UAA backbone (needing only p88 in *trans*). Finally, the RSE element could be under dynamic regulation through the folding of the alternative Basal structure that encompasses the Ff element within the p28 coding region, as mutating or deleting Ff appeared to also affect the robustness of TCV replication. Again, the Basal structure appears to be less critical when the replicon genome produces its own p28, as the m2-1 mutant would be expected to have disrupted the FfFa stem extensively, yet could replicate to 70% of its 813UAA parent (Figure 2).

Finally, we wish to address why the replication of TCV mutants with *trans*-provided p28 and p88 was inefficient compared to that of TCV replicons encoding their own p28 and p88. Multiple factors could have contributed to this inefficiency. First, both p28 and p88 were shown previously to repress TCV replication when overexpressed [10,12]. Assuming the heterogeneity of their expression in different cells, it is possible that complementation might have occurred in a fraction of cells where the threshold for repression was not reached. It is also possible that efficient replication requires these two proteins to be present at specific intracellular concentrations and/or ratio, or be produced in a temporarily regulated manner. These conditions would be difficult to meet with our experimental setup. Lastly, *cis*-production of these replication proteins may indeed be favored by the replication process [21,22]. Nevertheless, our new approach did allow for the revelation of novel translation-independent roles of several RNA secondary structures within the p28/p88 coding sequence. This approach may prove valuable for the examination of similar RNA structures in other viruses, leading to a better appreciation of the role played by these structures.
